# Patients prefer human psychiatrists over chatbots: a cross-sectional study

**DOI:** 10.3325/cmj.2025.66.13

**Published:** 2025-02

**Authors:** Goran Arbanas, Ante Periša, Ivan Biliškov, Jelena Sušac, Mirela Badurina, Dahna Arbanas

**Affiliations:** 1 Department for Forensic Psychiatry, Vrapče University Psychiatric Hospital, Zagreb, Croatia; 2 University Psychiatric Hospital Vrapče, Zagreb, Croatia; 3 Codeasy, Split, Croatia; 4 BHIDAPA, Sarajevo, Bosnia and Herzegovina; 5 Department for Diagnostics and Intensive Care, Vrapče University Psychiatric Hospital, Zagreb, Croatia; 6 Karlovac Pharmacies, Karlovac, Croatia

## Abstract

**Aim:**

To rate the level of patients’ satisfaction with responses on questions regarding mental health provided by human psychiatrists, pharmacists, and chatbot platforms.

**Methods:**

This cross-sectional study enrolled 89 patients who were pharmacologically treated for their mental disorder in one institution in Croatia and one in Bosnia and Herzegovina during October 2023. They asked psychiatrists, pharmacists, ChatGPT, and one Croatian chatbot questions about their mental disorder and medications and rated the satisfaction with the responses.

**Results:**

Almost half of the patients had used ChatGPT before the study, and only 12.4% had used the Croatian platform. The patients were most satisfied with the information provided by psychiatrists (4.67 out of 5 about mental disorder and 4.51 about medications), followed by pharmacists (3.94 about medications), ChatGPT (3.66 about mental disorder and 3.45 about medications), and the Croatian platform (3.66 about mental disorder and 3.44 about medications). Almost half of the participants believed it was easier for them to put a question to a psychiatrist than to a chatbot, and only 10% claimed it was easier to ask ChatGPT.

**Conclusion:**

Patients with mental health disorders were more satisfied with responses from their psychiatrists than from chatbots, and satisfaction with chatbots’ knowledge on mental disorders and medications was still too low to justify their usage in these patients.

The use of artificial intelligence (AI) in psychiatry dates back to the 1960s, when computer programs were first used to support diagnostic decisions and create treatment plans (1). Over the following decades, AI has been employed to diagnose depression, identify suicide ideation, predict suicidality from unstructured text, and forecast depression from social media posts data, to mention only a few applications (2-11). AI platforms on mobile devices enhanced medication adherence better than directly observed therapy in persons with schizophrenia, and smartphone sensor data were able to distinguish between patients with and without depression (12). Accuracy rates ranged from 62% to 98%, with lower values observed for smartphone data and higher values for physical functions and sociodemographic data (12). Furthermore, AI was used in neurofeedback therapy (13).

Chat Generative Pre-trained Transformer (ChatGPT) can provide clear, accurate, and detailed information about schizophrenia, alcohol-related disorders, attention deficit/hyperactivity disorder, and other mental disorders (14,15). It has successfully passed the United States Medical Licensing Examination and can provide differential diagnoses from clinical vignettes with 93% accuracy (14). ChatGPT’s answers to psychiatry questions had very good accuracy, completeness, and nuance (8 out of 10) (16). Furthermore, ChatGPT is available 24/7 and can provide companionship at any time (17). The use of specially trained chatbots (eg, Woebot and Tess) led to a reduction in depression and anxiety symptoms (18,19). ChatGPT least successfully diagnosed personality disorders (20) and was only moderately able to diagnose post-childbirth posttraumatic stress disorder, but managed better after additional training (21). In one study, 78% of participants were willing to use ChatGPT for self-diagnosis (22). They were willing to receive health advice from a chatbot, especially regarding logistical issues and preventative care, but less willing to receive diagnostic and treatment advice. They were only weakly able to distinguish between ChatGPT and human-generated responses, with the ability to identify the source of answers ranging from 49% to 86% (22). Some patients are concerned about being judged by their therapists and prefer to share sensitive and confidential information with a chatbot (18,23).

ChatGPT was not trained on medical data, and there are medically trained chatbots, such as Med-PaLM, clinical BERT, and BioGPT (21,22). However, additionally trained ChatGPT can provide non-judgmental and supportive responses. It was able to ask clarifying questions, provided feedback, suggested potential coping strategies, and encouraged patients to explore underlying triggers (19).

Human-chatbot relationship evolved from being superficial at the onset to being substantial and rewarding for the users (24). Unfortunately, despite ample opportunities to use chatbots in everyday practice, the involvement of psychiatrists has been low due to their lack of training in computer and information sciences (23).

While AI-based tools, including chatbots, are capable of providing accurate, guideline-consistent information, it is unclear whether they are able to offer emotional support and build a therapeutic alliance (25). Furthermore, some patients may prefer the judgment-free, always-available nature of AI-based chatbots, whereas others place greater value on the empathy, warmth, and nuanced understanding offered by humans.

Previous research has primarily focused on the satisfaction of professionals, such as psychiatrists, with chatbot responses. However, no study has yet examined patients’ satisfaction with chatbot-provided responses related to mental health. The primary aim of the study was to compare the level of patient satisfaction with responses provided by human psychiatrists, pharmacists, and chatbot platforms (ChatGPT and a Croatian platform Pomozi) regarding mental health disorders and treatments. The secondary aims were to explore the factors influencing patient satisfaction, including sociodemographic characteristics, diagnosis type, and platform-specific features; and to assess the extent to which chatbots were perceived as professional, knowledgeable, and easy to approach compared with human providers.

## Participants and methods

This cross-sectional study involved patients who visited the outpatient clinics of Vrapče University Psychiatric Hospital in Zagreb, Croatia, and BHIDAPA clinic in Sarajevo, Bosnia and Herzegovina, from October 1 to October 31, 2023. Of 121 patients with internet access, 89 agreed to participate. Only patients with a disorder from the ICD-10 code F were enrolled. Inpatients were not included because we believe that outpatients better represent people with mental disorders, and many hospitalized patients have thought disturbances or psychotic symptoms that may influence their understanding of the study. Furthermore, the topic of AI may induce delusional ideas in these patients. The study was approved by the Ethics Committee of the Vrapče University Psychiatric Hospital.

Patients were offered to complete either a paper or an online version of the questionnaire (Supplemental Material(Supplementary Material)). The author-created questionnaire consisted of two parts. The first part gathered information on sociodemographic data: gender, age, marital status, education, working status, socioeconomic status (on a Likert scale), and age of first contact with a psychiatrist. The second part inquired about satisfaction with the information given by the chatbots. Patients were instructed to ask ChatGPT and the Pomozi chatbot (www.pomozi.hr) the same questions about their disorder and medications that they asked their psychiatrist or pharmacist. The patients were asked to rate on a 5-point Likert scale how satisfied they were with information obtained from each source (1 – not at all satisfied; 2 – a bit dissatisfied; 3 – neither satisfied nor dissatisfied; 4 – satisfied; 5 – very satisfied). They were also asked with whom they found it easier to ask about their mental problems.

The questionnaire underwent content validation through an expert review. A panel of experts in psychiatry and computer science assessed the items for clarity, relevance, and comprehensiveness.

### Statistical analysis

Categorical data are expressed as counts and percentages, while continuous data are presented as means with standard deviations. Normality of continuous data was assessed with a Shapiro-Wilk test. Differences in satisfaction scores between the groups were assessed with a χ^2^ test for categorical variables and a paired-samples *t* test or a Wilcox signed-rank test for continuous variables. Finally, to assess the correlations between sociodemographic and diagnostic variables and the level of satisfaction, ordinal regression analysis was performed. Statistical analysis was conducted using SPSS, version 26.0 (IBM Corp., Armonk, NY, USA).

## Results

The study enrolled 89 participants (60.7% women) with a mean (±standard deviation) age of 40.1 ± 12.4 years. Most were employed (67.4%) and had an average socioeconomic status (51.7%), with nearly half (47.2%) having completed college. The primary diagnoses were neurotic, stress-related, and somatoform disorders (36.3%), schizophrenia-related disorders (31.3%), and mood/affective disorders (21.3%), with fewer cases of organic mental disorders (3.8%), substance use disorders (3.8%), behavioral syndromes (1.3%), and personality disorders (2.5%) (Table 1, Table 2). Almost half of the sample (47.2%) had used ChatGPT before this study, and only 12.4% had used the Pomozi platform. Before the study, only 12.6% and 2.2% used ChatGPT and Pomozi, respectively, to ask questions about their mental disorders. No significant differences in satisfaction responses were observed between participants who had previously used the platforms and those who had not.

**Table 1:** Sociodemographic characteristics of the respondents (N = 89)

	Whole sample (N = 89)	Men (N = 35)	Women (N = 54)	
Age	40.1 ± 12.4	38.5 ± 12.9	41.1 ± 12.2	*t* = -0.947; *P* = 0.347
Married/in a relationship				χ^2^ = 0.681; *P* = 0.409
yes	30	10	20	
no	59	25	34	
Employed				χ^2^ = 6.712; *P* = 0.010
yes	60	18	42	
no	29	17	12	
Socioeconomic status				χ^2^ = 0.993; *P* = 0.609
below average	15	6	9	
average	46	16	30	
above average	28	13	15	
Education				χ^2^ = 13.827; *P* = 0.001
primary	5	4	1	
secondary	42	23	20	
college	42	8	33	

**Table 2:** Participants’ diagnoses

Section in ICD-10	Number of participants	%
F0 Organic, including symptomatic, mental disorders	3	3.8
F1 Mental and behavioral disorders due to psychoactive substance use	3	3.8
F2 Schizophrenia, schizotypal and delusional disorders	25	31.3
F3 Mood /affective/ disorders	17	21.3
F4 Neurotic, stress-related and somatoform disorders	29	36.3
F5 Behavioral syndromes associated with physiological disturbances and physical factors	1	1.3
F6 Disorders of adult personality and behavior	2	2.5

Participants reported the highest mean satisfaction with the responses provided by their psychiatrists (4.67 about mental disorders and 4.51 about medications) followed by pharmacists (3.94 about medications), ChatGPT (3.66 about mental disorders and 3.45 about medications) and Pomozi (3.66 and 3.44, respectively) (*P* < 0.01, Table 3). There were no significant differences in the satisfaction ratings between the two platforms and between the sexes.

**Table 3:** Satisfaction of psychiatric patients with answers about their mental disorder and medications from their psychiatrists, pharmacists, ChatGPT, and the Pomozi platform

	Satisfaction score (mean ±SD, out of 5) with responses on questions about	
	mental disorder	medications
Psychiatrists	4.67 ± 0.64	4.51 ± 0.88
Pharmacists	-	3.94 ± 1.10
ChatGPT	3.66 ± 1.07	3.45 ± 1.03
Pomozi	3.66 ± 1.02	3.44 ± 1.11
Comparisons		
Psychiatrists vs pharmacists	-	*t* = 3.704; *P* < 0.001
Psychiatrists vs ChatGPT	*t* = 7.359; *P* < 0.001	*t* = 7.089; *P* < 0.001
Psychiatrists vs Pomozi	*t* = 7.668; *P* < 0.001	*t* = 6.814; *P* < 0.001
Pharmacists vs ChatGPT	-	*t* = 2.991; *P* = 0.003
Pharmacists vs Pomozi	-	*t* = 2.919; *P* = 0.004
ChatGPT vs Pomozi	*t* = 0; *P* = 1	

We also assessed the correlations between satisfaction with responses from different sources and sociodemographic data. There was only a very weak correlation between age and satisfaction with psychiatrists’ responses about mental disorders and medications (r = 0.25 and r = 0.25, respectively), meaning that older participants slightly more positively assessed the responses. There was also a very weak negative correlation between socioeconomic status and satisfaction with responses about medications from psychiatrists and pharmacists (r = -0.24 and r = -0.20, respectively), meaning that people with lower socioeconomic status had slightly higher satisfaction with the responses from psychiatrists and pharmacists.

Nearly half of the participants reported that it was easier to put a question to their psychiatrists than to ChatGPT or Pomozi (48.9% and 45.2%, respectively). Only 12.5% and 7.1% of participants, respectively, said it was easier to talk to ChatGPT or Pomozi. None of the participants believed that the platforms were more professional and knowledgeable than their psychiatrists.

Statistical analysis according to the diagnosis was performed only for psychotic disorders, mood disorders, and anxiety/stress-related disorders, because of the small number of patients in the other groups. The only significant difference was that patients with mood disorders were more satisfied with the answers from psychiatrists than patients with anxiety/stress-related disorders.

## Discussion

In this study, patients with mental health disorders showed greater satisfaction with the answers from psychiatrists more than from chatbots. Almost half of our participants had used ChatGPT before the study and 12% had used Pomozi, a Croatian chatbot that was not advertised and could not be found on internet search engines. It is not surprising that a high percentage of patients used modern technologies. A Croatian study from 2022 showed that about 70%-90% of Croatian patients with schizophrenia and depression searched the internet for information on mental health (26). It is only somewhat unexpected that a small minority of our participants used ChatGPT to ask questions about their mental disorders.

In previous research, professionals, including psychiatrists, evaluated ChatGPT’s answers as accurate, complete, nuanced, and in alignment with professional guidelines (27). ChatGPT provided better answers than did psychiatrists using ChatGPT and especially psychiatrists using other sources (16). Unlike psychiatrists, our participants were not as enthusiastic about chatbots. They rated the responses from two platforms with a score of 3.5 out of 5, being more satisfied with answers from psychiatrists and pharmacists. This agrees with the results from healthy participants in other studies, who graded ChatGPT similarly (3.9 for logistical questions; 3.5 for preventative care; 2.9 for diagnostics, and 2.9 for treatment) (22). Although ChatGPT and other chatbots give very precise and accurate responses and follow professional guidelines, patients may need more support and relationship. Studies highlight the importance of therapeutic alliance, warmth, and empathy in fostering positive outcomes in psychotherapy. Chatbots may partially emulate these therapeutic factors by providing non-judgmental support and forming relationships through phases of exploration, affect, and stability (10,15,26).

According to the technology acceptance model (TAM), users' acceptance of technology is influenced by perceived usefulness and ease of use (28,29). In our study, patients may have had lower satisfaction with chatbots as they perceived them as less useful, particularly when it came to providing the emotional support and empathy critical for mental health care. Similarly, perceived ease of use could explain the reluctance to engage with chatbots if patients found them less intuitive or trustworthy. Another reason may be that the patients did not have enough time to develop a relationship with the chatbots as they were instructed to have only one interaction with the platforms.

We did not observe a strong correlation between sociodemographic characteristics and satisfaction with responses from psychiatrists, pharmacists, and platforms. Expectedly, older patients more positively assessed psychiatrists’ responses. This might be due to the fact that young adults are more open to AI as they have been exposed to the internet and electronic communication devices from an early age (30).

Interestingly, respondents rated similarly ChatGPT and Pomozi. We expected the Pomozi platform to perform better because it is a Croatian-language platform specifically designed to provide psychological support. Nevertheless, ChatGPT can speak any language, and possible language nuances (if any) made no difference (15,31-33).

The main limitation of the study is that the sample size was not determined based on a formal power analysis. Yet, a *post-hoc* power analysis for the comparison of satisfaction scores, based on the observed large effect size (d = 1.10) and total sample size (N = 89), yielded a statistical power of 0.999, indicating that this analysis was adequately powered. However, analyses with smaller effect sizes might not have achieved sufficient power. Furthermore, with a larger sample it would be possible to detect differences among people with different mental disorders. Also, the results cannot be generalized to people with mental disorders in other regions and countries. Although participants were recruited in the largest psychiatric institution in Croatia, they may not represent the broader population of patients with mental disorders, which might have led to selection bias. Future studies should include a more diverse range of settings to enhance generalizability. Furthermore, data collection was based on self-reported satisfaction, which is inherently subjective and can introduce response bias. Future research should incorporate independent assessments or observational methods to validate findings. Finally, although the questionnaire underwent content validation, it did not involve formal psychometric validation, such as construct or criterion validation.

In conclusion, this was the first study of the attitudes of patients with mental disorders toward ChatGPT responses, which showed that patients in Croatia and Bosnia and Herzegovina believed that psychiatrists were better sources of information than chatbots.
