# Supplementary material for: Patients prefer human psychiatrists over chatbots: a cross-sectional study
Source: Croat Med J. 2025 Feb;66(1):13–9. doi: 10.3325/cmj.2025.66.13 (PMC11947973; doi:10.3325/cmj.2025.66.13)
Supplement: Supplementary Material [file CroatMedJ_66_s001.pdf]

Supplementary material

The questionnaire

By filling in this questionnaire, you participate in research of the satisfaction of people with mental health problems with information about their disorder and their treatment patients with mental health issues get from their physicians or pharmacists and with the satisfaction with information that can be found on the Internet.

Gender:

Age:

Marital status:

Educational level:

1 elementary school

2 secondary school

3 bachelor’s degree

4 master’s degree or higher

Are you employed? YES NO

How would you compare your socioeconomic status with the average family in Croatia/Bosnia and Herzegovina?

1 much worse

2 a bit worse

3 the same

4 a bit better

5 much better

When did you contact a psychiatrist for the first time?

List five things you use internet most often for!

Have you ever before heard about ChatGPT? YES NO

What have you used ChatGPT so far for?

Have you ever used ChatGPT for answering questions about your mental health issues? YES NO

Have you ever asked advice from ChatGPT related to your mental health issues? YES NO

Have you ever asked advice from ChatGPT about your medications used for your mental health issues? YES NO

Have you ever before heard about the www.pomozi.hr platform? YES NO

Have you ever used this platform for answering questions about your mental health issues? YES NO

Have you ever asked advice from the platform related to your mental health issues? YES NO

Have you ever asked advice from the platform about your medications used for your mental health issues? YES NO

How satisfied are you with information given by your psychiatrist about your mental disorder?

1 not at all satisfied
2 a bit dissatisfied
3 not satisfied, nor dissatisfied
4 satisfied
5 very satisfied

How satisfied are you with information given by your psychiatrist about the medication you are using?

1 not at all satisfied
2 a bit dissatisfied
3 not satisfied, nor dissatisfied
4 satisfied
5 very satisfied

How satisfied are you with information given by the pharmacist about the medication you are using?

1 not at all satisfied
2 a bit dissatisfied
3 not satisfied, nor dissatisfied
4 satisfied
5 very satisfied

We kindly ask you to visit the platform www.pomozi.hr and ask questions about your mental health issues and medications you are using (trying to ask the same questions you asked your psychiatrist and/or pharmacist).

Do the same with visiting ChatGPT. Do enter this platform you need to register at OpenAI. Ask the same questions you posed to www.pomozi.hr. After you do this, please answer the following questions.

In case of any dilemmas or questions about the advice given by the platforms you can contact us.

How satisfied are you with information given by ChatGPT about your mental disorder?

1 not at all satisfied

2 a bit dissatisfied

3 not satisfied, nor dissatisfied

4 satisfied

5 very satisfied

How satisfied are you with information given by ChatGPT about the medication you are using?

1 not at all satisfied

2 a bit dissatisfied

3 not satisfied, nor dissatisfied

4 satisfied

5 very satisfied

Have you got some new information from ChatGPT that you did not receive from your psychiatrist?

YES NO

If yes, what was it?

Compared to your psychiatrist, how would you rate the easiness of asking questions about your mental health to ChatGPT?

1 it was much easier to ask ChatGPT

2 it was a bit easier to ask ChatGPT

3 it was the same

4 it was easier to ask a psychiatrist

5 it was much easier to ask a psychiatrist

Please rate the answers given by ChatGPT:

1 ChatGPT gives much more professional answers compared to my psychiatrist

2 ChatGPT gives more professional answers compared to my psychiatrist

3 Answers given by ChatGPT and my psychiatrist are of the same professional level

4 My psychiatrist gives more professional answers compared to ChatGPT

5 My psychiatrist gives much more professional answers compared to ChatGPT

What you think is the most important advantage of ChatGPT?

What you think is the most important disadvantage of ChatGPT?

How satisfied are you with information given by the platform www.pomozi.hr about your mental disorder?

1 not at all satisfied

2 a bit dissatisfied

3 not satisfied, nor dissatisfied

4 satisfied

5 very satisfied

How satisfied are you with information given by the platform www.pomozi.hr about the medication you are using?

1 not at all satisfied

2 a bit dissatisfied

3 not satisfied, nor dissatisfied

4 satisfied

5 very satisfied

Have you got some new information from the platform that you did not receive from your psychiatrist?

YES NO

If yes, what was it?

Compared to your psychiatrist, how would you rate the easiness of asking questions about your mental health to the www.pomozi.hr platform?

1 it was much easier to ask the platform

2 it was a bit easier to ask the platform

3 it was the same

4 it was easier to ask a psychiatrist

5 it was much easier to ask a psychiatrist

Please rate the answers given by the www.pomozi.hr platform:

1 The platform gives much more professional answers compared to my psychiatrist

2 The platform gives more professional answers compared to my psychiatrist

3 Answers given by the platform and my psychiatrist are of the same professional level

4 My psychiatrist gives more professional answers compared to the platform

5 My psychiatrist gives much more professional answers compared to the platform

What you think is the most important advantage of the www.pomozi.hr platform?

What you think is the most important disadvantage of the www.pomozi.hr platform?
